# Influence of sustainable waste granite, marble and nano-alumina additives on ordinary concretes: a physical, structural, and radiological study

**DOI:** 10.1038/s41598-024-72222-4

**Published:** 2024-09-24

**Authors:** Alaa A. Mahmoud, Alaa A. El-Sayed, Ayman M. Aboraya, Islam N. Fathy, Mohamed A. Abouelnour, Islam M. Nabil

**Affiliations:** 1 Civil Engineering Department, Faculty of Engineering, Fayoum University, Fayoum, Egypt; 2 Construction and Building Engineering Department, October High Institute for Engineering & Technology, Giza, Egypt; 3 Construction and Building Engineering Department, Higher Institute of Engineering, Culture & Science City, Giza, Egypt; 4 Physics Department, Faculty of Science, Fayoum University, Fayoum, Egypt

**Keywords:** Waste granite, Marble, Concrete, Physical/structural properties, MCNP, Radiation shielding, Materials science, Physics, Applied physics, Nuclear physics

## Abstract

This study investigates the individual and combined effects of enhancing the radiation shielding properties of waste concrete using the optimal mix design of two waste material powders of different compositions. Marble (MD) and granite (GD) waste dust were individually utilized as partial replacements for cement at a replacement ratio of 6%. Furthermore, two additional mixes were prepared by incorporating 1% by cement weight of nano alumina (NA) to enhance the microstructure of the studied waste concrete. The MGA-concrete was analyzed using X-ray Fluorescence, Energy dispersive X-ray, X-ray diffraction analysis, transmission electron microscopy, and scanning electron microscope techniques. The radiation shielding assets of the examined Concrete samples, such as the linear attenuation coefficient (μ), half value layer (H_1/2_), tenth value layer (T_1/10_), and fast neutron removal cross-section were evaluated using the MCS5 Monte Carlo simulation algorithm and Phy-X software. The results showed that the linear attenuation for the GMN-concretes’ order is CO < MD < GD < NA < MD + NA < GD + NA. The GD + Na concrete sample presents the best neutron performance. The studied GMN-concrete samples provide the best protection against γ-rays and fast neutrons. Lastly, the excellent performance of the mixes of waste Granite, Marble, and Nano-Alumina on ordinary would pave the way for their employment as radiation shielding in various nuclear and medical facilities.

## Introduction

These days, many industrial, medical, and nuclear settings use machines that make artificial ionizing radiation, like X-rays and γ-rays. This is possible because of advances in technology ^1–3^. But, cancer, nausea, vomiting, and, in extreme cases, death, are some of the negative health effects that can result from long-term and excessive exposure to this radiation ^4,5^. Tissue water molecules become ionized when high-energy photons interact with them. Because it damages DNA's outside and inside, this ionization is dangerous to DNA ^6^. The environment, animals, and humans are all at risk from gamma/X-rays and neutrons. Finding better materials for radiation attenuation and shielding is an intriguing topic for many researchers for these reasons ^7–10^. It is well knowledge that shielding and attenuation materials can reduce the amount of radiation that workers are exposed to by establishing a barrier between the radiation sources and the location where they are located or the workers themselves.

In order to reduce the negative effects of radiation, one of the fundamental concepts of radiation protection is the selection of proper shielding materials. This is an essential step in the process. It is still a challenge for researchers to gain a handle on this particular aspect. Research on the radiation-shielding capabilities of a wide variety of materials, such as synthetic and natural rubber, concrete, brick, metal, and polymers, may be found in abundance in the published literature ^11–14^. By utilizing this knowledge, engineers are able to develop cutting-edge materials that exhibit specific traits and capabilities that were previously unreachable within the area of material science. These materials are able to be created by applying this information. Once that was accomplished, these materials would be utilized in a wide range of technological sectors worldwide. When it comes to selecting radiation shielding materials, the most important factor to take into account is the ability of different materials to offer protection against ionizing radiation. Particles of cement, borate glasses, fibers, and polymers are some examples of the types of materials that can be utilized to provide protection against radiation ^15^. The complexity of these materials can range from simple to quite complex ^16–18^. The ability of a material to absorb or diminish radiation is a primary consideration in the selection of materials for radiation attenuation. Lead and concrete are two materials that are frequently used because of their capacity to either absorb or distribute radiation. Among other things, boron has the ability to absorb neutrons ^19–21^. Materials are chosen considering exposure rate reduction, source kind, physical constraints, and cost-effectiveness. Shielding applications in nuclear engineering extensively use gypsum, lead, glass, and cement ^22–24^. Direct shielding applications and nuclear power plant construction use these materials ^25,26^. Magnesium borosilicate glasses exhibit superior radiation shielding capabilities compared to traditional glass materials. Increasing ZnO content in the composite enhances neutron shielding properties. A novel glass system composed of 57.6% TeO_2_, 38.4% ZnO, and 4% NiO has been developed and evaluated for its radiation shielding capabilities ^27^. The construction sector is significant in global resource extraction, consuming 40% of materials and contributing 50% of global greenhouse gas emissions ^28^. As a consequence of this, construction operations result in the generation of significant quantities of garbage, which frequently finds its way into landfills. One of the most important components of the building industry is the manufacture of concrete, which is a construction material. The production process, on the other hand, requires a significant number of resources, with aggregates, cement, and water being the primary components. The output of cement around the world has increased, and it is projected to reach 4.4 billion tons in 2022. This is a 175% spike in production since the beginning of the third millennium ^29^. This production process is associated with substantial carbon dioxide emissions, with approximately 1 ton of CO2 released for every ton of ordinary portland cement produced. Cement manufacturing contributes about 7% of global annual carbon dioxide emissions, positioning it as the third largest industrial emitter^30^. As an alternative to clinker or as direct mineral additives in concrete, supplementary cementitious materials (SCMs) such fly ash, calcined clays, slags, and natural pozzolans are utilized in order to mitigate the impact that this industry on the environment ^31^. The current study represents an extension of a previously published study ^32^, where the authors utilized varying proportions of marble and granite powders as partial replacements for cement while investigating their effects on concrete's mechanical, physical, and microstructural properties. The results revealed a significant improvement in all properties of the produced waste concrete with varying replacement ratios, with a relative superiority observed for the optimal replacement ratio at 6% for both Marble dust (MD) and Granite dust (GD). Additionally, the practical program involved harnessing the capabilities of nanotechnology by incorporating varying proportions of Nano alumina (NA) into the concrete mixes, where the results demonstrated that adding 1% by weight of NA is the optimum ratio. The second phase of the experimental program involved optimizing the results by using the optimal replacement ratios for both MD and GD, along with a 1% addition of NA. This improved the resulting concrete's mechanical properties and gave it a denser and more compact microstructure. Through the current study, the previously tested optimal replacement ratios were experimented with to investigate the impact of using waste materials and NA on the concrete's shielding properties to various radiation forms. The use of NA at small replacement ratios as a substitute for cement has shown the ability to enhance the concrete's shielding to gamma-ray penetration ^33^. The reason for this is that it has the capacity to enhance the internal microstructure of concrete, so making it denser and more compact. This, in turn, disrupts the paths via which radiation is able to infiltrate. In spite of the fact that the effects of marble and granite powders are being investigated for the first time in radiation shielding concretes, the results of the literature that have been reported on the notable positive impacts on the microstructural level and density of the concrete that is produced at optimal usage ratios are encouraging in this regard. This notion is further confirmed by a number of earlier studies that have proven a significant improvement in the radiation resistance qualities of concrete, particularly with regard to gamma radiation, when materials with chemical and mineral compositions that are comparable to those of waste powders made from marble or granite are utilized ^34–36^.

This work investigates the individual and combined effects of enhancing the radiation shielding properties of waste concrete using the optimal mix design of two waste material powders of different compositions. MD and GD were individually utilized as partial replacements for cement at a replacement ratio of 6%. Furthermore, two additional mixes were prepared by incorporating 1% by cement weight of NA to enhance the microstructure of the studied waste concrete. The MGA-concrete was analyzed using X-ray Fluorescence (XRF), Energy dispersive X-ray (EDX), X-ray diffraction analysis (XRD), Transmission Electron Microscopy (TEM), and Scanning Electron Microscope (SEM) techniques. The radiation shielding assets of the examined Concrete samples, such as the linear attenuation coefficient (μ), half value layer (H_1/2_), and tenth value layer (T_1/10_), were evaluated using the MCS Monte Carlo simulation algorithm and Phy-X software. The MCS code was also used to determine the fast neutron removal cross-section (GMN_FNRCS_), their half-value length (GMN_HVLFNRCS_), and relaxation length (GMN_λFNRCS_). Most of the studied radiation attenuation parameters were compared to some commonly used glasses and concretes.

## Materials and mix design

### Materials properties

#### Cement, aggregates, and water

During the course of this investigation, the conventional ordinary Portland cement (OPC) type I grade 52.5 N was utilized. The average particle size of this cement was 9 μm, and it adhered to the specifications set forth by ASTM C 150 ^37^. The cement that was used has a specific gravity of 3.15 and a surface area of 3500 cm^2^/g. The information regarding the chemical composition of the cement is presented in Table 1, which allows for easier comprehension. Sand that was naturally siliceous was used as the fine aggregate, while crushed dolomite with a maximum nominal size of 19 mm was used as the coarse aggregate. For the purposes of the concrete mixing and curing procedures, fresh, clean water that satisfies the requirements indicated in ASTM C1602/C1602M 37 was utilized ^38^.

**Table 1:** Chemical composition of the used powders (% weight).

Type	SiO_2_	CaO	Al_2_O_3_	Fe_2_O_3_	MgO	K_2_O	Na_2_O	SO_3_	P_2_O_5_	Cl	TiO_2_	LOI*
Granite	66.500	2.840	14.700	3.220	0.830	6.160	3.130	0.090	0.320	0.040	0.450	1.420
Marble	0.590	55.600	0.110	0.030	0.290	0.020	0.010	0.020	–	0.020	–	43.200
Cement	19.640	63.230	4.400	3.340	1.240	0.170	0.200	3.070	0.130	0.090	0.340	4.050
Nano alumina	0.020	–	99.100	0.020	–	–	0.060	–	–	–	–	0.700

*LOI: Loss on Ignition at 1000 °C.

#### Waste material powders

**Fig. 1:**
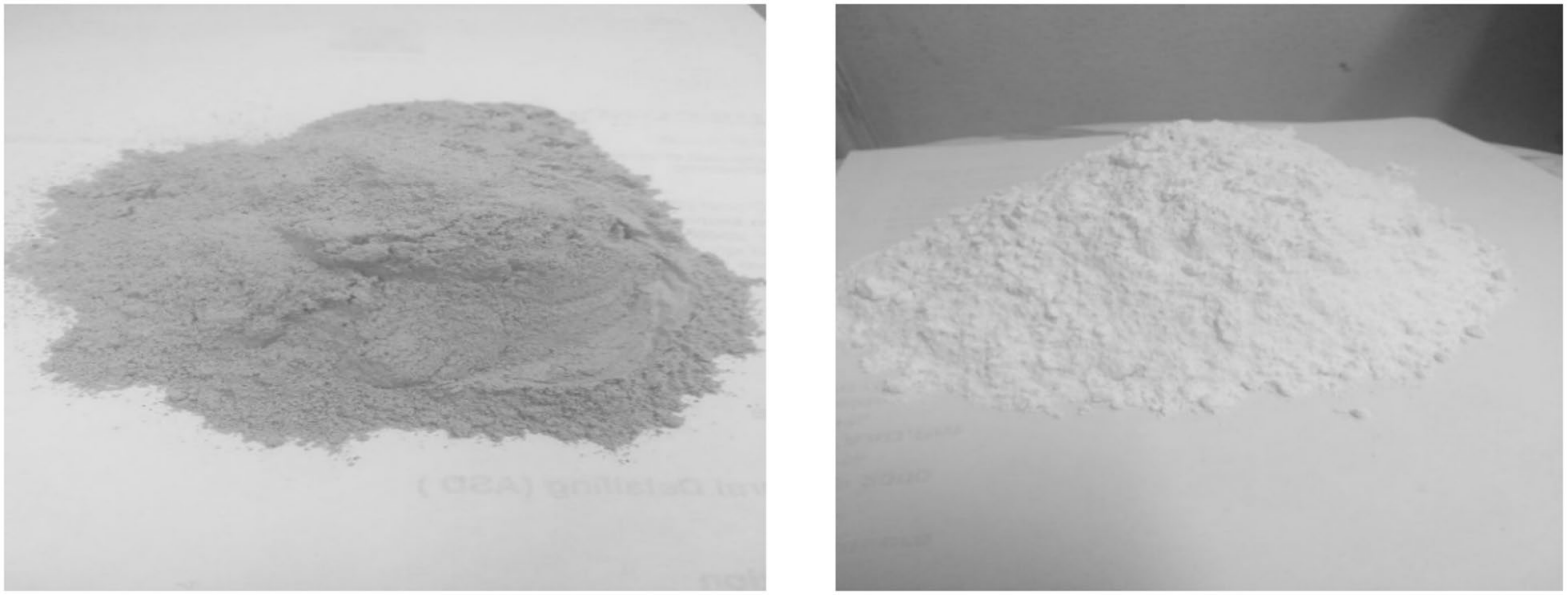
Granite and marble dusts after preparation.

The marble and granite dust were sourced from ornamental stone factories in the Shaq El-Thu’ban area, Egypt, representing a by-product generated during the cutting, shaping, and polishing processes of Karara marble and red granite. Initially present in a wet state, the dust dried at 100 °C for two hours in a laboratory oven to ensure consistent W/C ratios within the concrete mixes. Subsequently, the dried dust was ground in a ball mill for ten minutes to eliminate agglomerates and then sieved through a #200 sieve to obtain particles with a size less than 75 microns, suitable for cement replacement. The comparison between marble and granite dusts reveals distinct characteristics. Marble dust, typically white, boasts a specific gravity of 2.64 and a surface area of 7600 cm^2^.g^−1^. Its average particle size (D50) is 5.1 µm. In contrast, granite dust exhibits a grayish hue, a slightly higher specific gravity of 2.73, and a significantly larger surface area of 14,650 cm^2^.g^−1^. Notably, granite dust particles are finer, with an average size (D50) of 2.5 µm. These differences in color, density, surface area, and particle size influence the potential applications of each material. The final appearance of the used waste powders after preparation is shown in Fig. 1.

#### Nano alumina

**Fig. 2:**
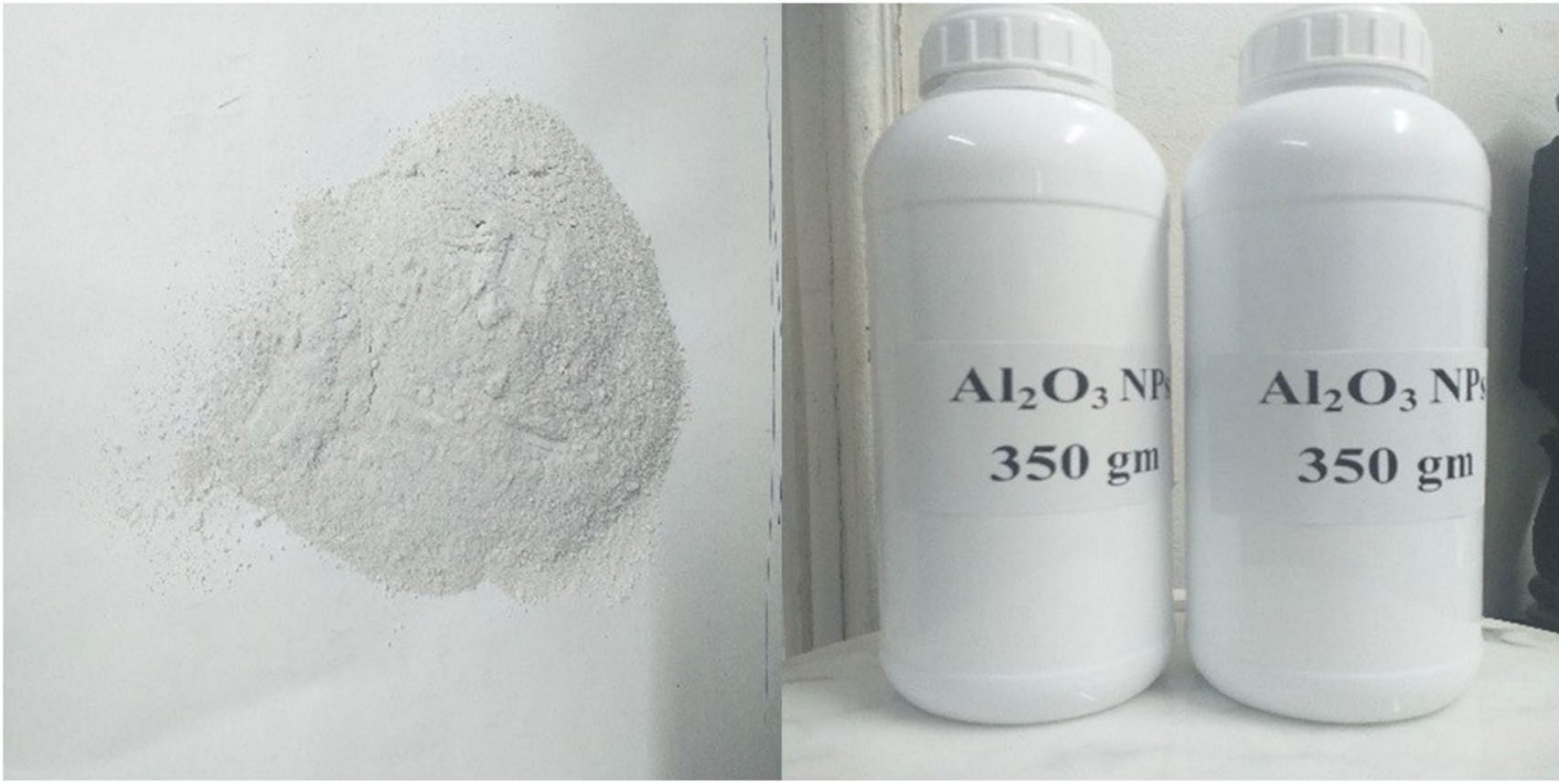
Nano alumina used in the work.

This investigation incorporates commercially available, high-purity (> 99%) spherical nano-alumina particles with an average diameter of 20 nm ± 5 nm. Notably, these nanoparticles (shown in Fig. 2) possess a substantial surface area of 175 m^2^.g^−1^ and density of 0.3 g.cm^−3^, characteristics that are anticipated to influence their effectiveness in diverse applications.

### Mix design

In addition to the control mix, five blends were designed to represent the individual and combined optimal replacement ratios of the investigated materials as partial replacements for cement. In the case of individual replacement, the ratios that achieved the best mechanical strength and improved microstructure of the resulting concrete in the literature research ^32^ were adopted for both MD, GD, and NA at 6%, 6%, and 1%, respectively. Additionally, two blends were prepared, each representing the optimal addition ratio of the waste material powders along with a 1% NA.

## Theoretic base

### γ-attenuation

The radiation performance of the materials can be described using a variety of attenuation coefficients which are as follows ^39–43^:

1 $${\text{I }} = {\text{ I}}_{{\text{o}}} {\text{e}}^{{ - \mu {\text{x}}}}$$

2 $$\mu_{{\text{m}}} = \frac{\mu }{\rho }$$

3 $${\text{H}}_{{{1}/{2}}} = \frac{{{\text{ln}}2}}{\mu }$$

4 $${\text{T}}_{{{1}/{1}0}} = { }3.32{\text{ H}}_{{{1}/{2}}}$$

5 $${\text{MFp}} = \frac{1}{\mu }$$

The symbol I represents the strength of the γ-rays that have entered the substance. All of the following are represented by Io: the mass attenuation (μm), the half-value layer (H_1/2_), the tenth value layer (T_1/10_), the mean free path (MFp), and the intensity of the main gamma without the material. The thickness of the material is represented by x, to the contrary.

### Neutron attenuation

The determination of the neutron attenuation potential of the materials that have been mentioned can be accomplished by computing the fast neutron removal cross-section (FNRCS) by employing the equation FNRCS =$\sum_{i}{W}_{i}$ x ρ ^44–46^. The sign ${W}_{i}$ is used to represent the partial density, while the symbol σ was used to represent the density of the material, and the subscript i was used to represent the mass-cross-section (σ) of the component ^47–49^. The half-value layer (HVL_FNRCS_) was determined using the formula. ${HVL}_{\text{FNRCS}} = \frac{ln2}{FNRCS}$, and the relaxation length (λ_FNRCS_) was calculated as $\frac{1}{FNRCS}$ ^18,50–53^.

## Characterizations

Methods such as XRF, EDX, XRD, TEM, and SEM were employed to investigate the microstructure, particle size, crystal structure, and mineralogical makeup of the dust in order to identify the various pure and mixed materials that comprise the GMN-Concrete samples system.

## Radiation shielding measurements

### MCNP code

**Fig. 3:**
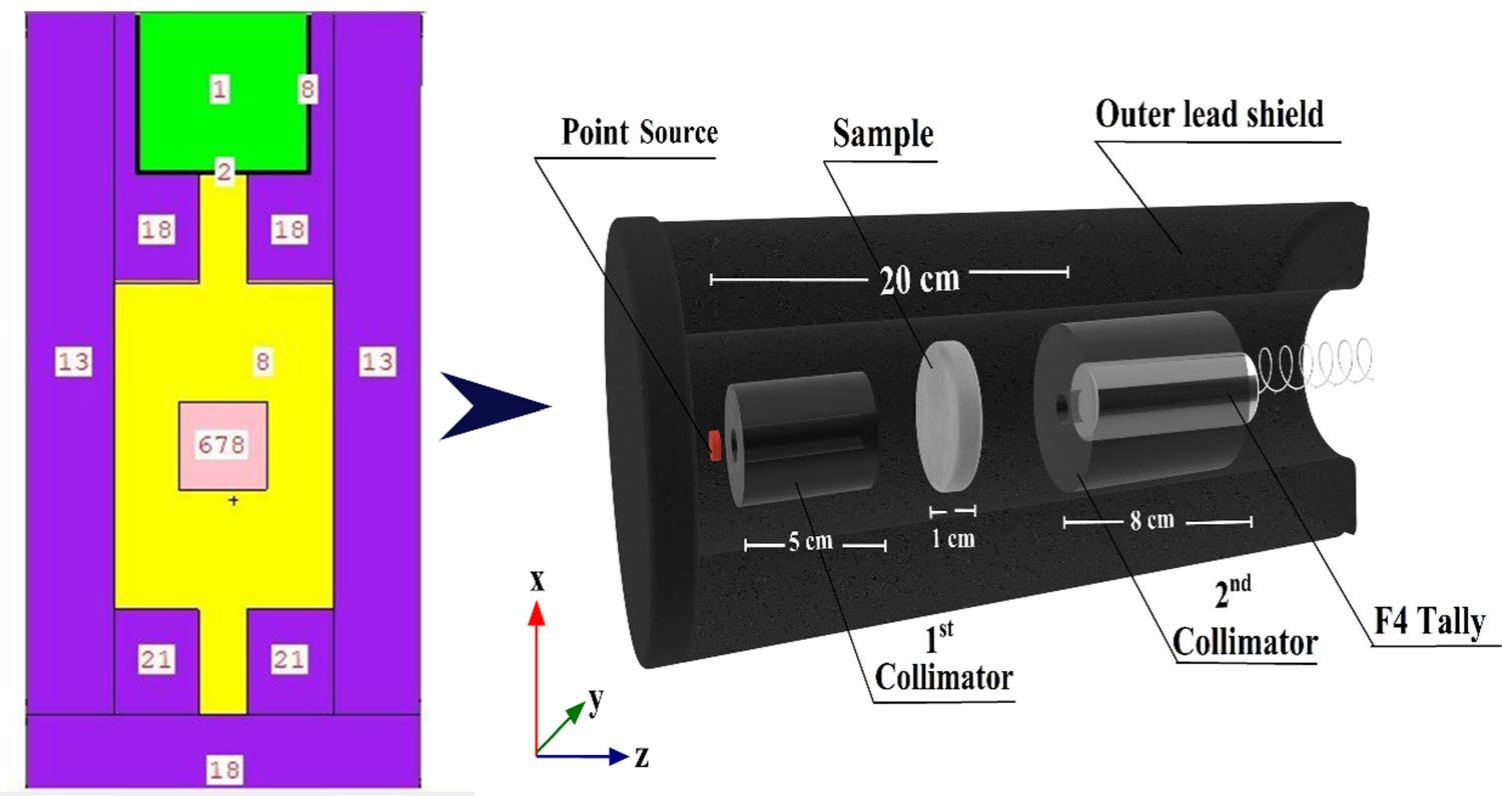
The energetic view of the radiation attenuation simulation used for the prepared GMN-concrete samples.

Using the Monte Carlo technique, the code is built to mimic real-world particles. In order to predict the theoretical strength of γ-rays released by γ-point sources, the MCS simulation system was employed. An energetic γ-emitter source operating in the photon energy range (Pγ) of 0.015 ≤ Pγ ≤ 15 in MeV was included in the simulation ^54,55^. A comparison of the γ-ray intensity before and after passing through the investigated glass materials was the intended objective. Radiation safety and shielding, dose calculation, detector design, and other research fields frequently favor MCS codes ^44,56–60^. Reasons for this preference include these codes' many useful features, such as fast calculations, adaptability to different geometrical designs, and operation across a wide range of energies. This method takes into account various photon interaction mechanisms and attempts to make electrons, neutrons, and γ-rays more mobile. Running an MCS simulation requires precise data on the geometry, source-to-detector distance, source dimensions, and chemical and elemental composition and densities of the concrete samples being examined. This data is found on the SDEF card ^61,62^. A predetermined two-dimensional and three-dimensional setup served as the basis for the development of the simulation's geometric configuration, which can be seen in Fig. 3. In accordance with the experimental system, each and every parameter has been recognized as being consistent. Text lines were used to generate the input files so that the MCS simulation could be run. There were six distinct components that comprised the cell, which included a radioactive source, a collimator for 1rays and 2rays γ-radiations, a sample with a cubic shape, and a detector. An SDEF mono γ-energetic flow was identified as a point source of γ-rays for every input file that fell within the range of 0.015 ≤ Pγ ≤ 15 MeV during the analysis. It has been established that the neutron source is a californium spectrum, which functions within the En ≤ 11 MeV range for the purpose of achieving rapid elimination σ attenuation. The specimens emerged in the shape of a cubic layer during the generation process. In addition to this, the densities and elemental compositions of the specimens that were examined were documented in the material card of the text lines. Within a lead collimator that was specifically designed for the 2ry γ-rays, the detector was effectively mounted. By using a Tally command, we may add up all the values from F4:P ^56,63^. At the same time, the F4:N method calculates the average duration of the γ-rays and neutrons that are emitted by simulated γ/neutron radiation sources. To protect the generated detector, collimators, source, and specimens under investigation, a lead outer shield was used. An Intel Core i5 was utilized in order to carry out the calculations. Multiple NPS (11^7^) attempts were carried out for every single file in order to guarantee that the random statistical errors remained below 1%. There were approximately thirteen minutes of total runtime for each of the calculations that involved a total of one hundred forty input files.

### Phy-X software

To confirm the accuracy of the MCS model ^55,64^, the Phy-X/PSD program was utilized. To determine the discrepancies (ϓ., %), the PhyX values for the concrete samples were compared with the MCS results.

6 $$\Upsilon \, \left( \% \right) \, = \left| {\frac{MCS - PhyX}{{MCS}}} \right| \times 100$$

## Results and discussion

### Samples characterization

**Fig. 4:**
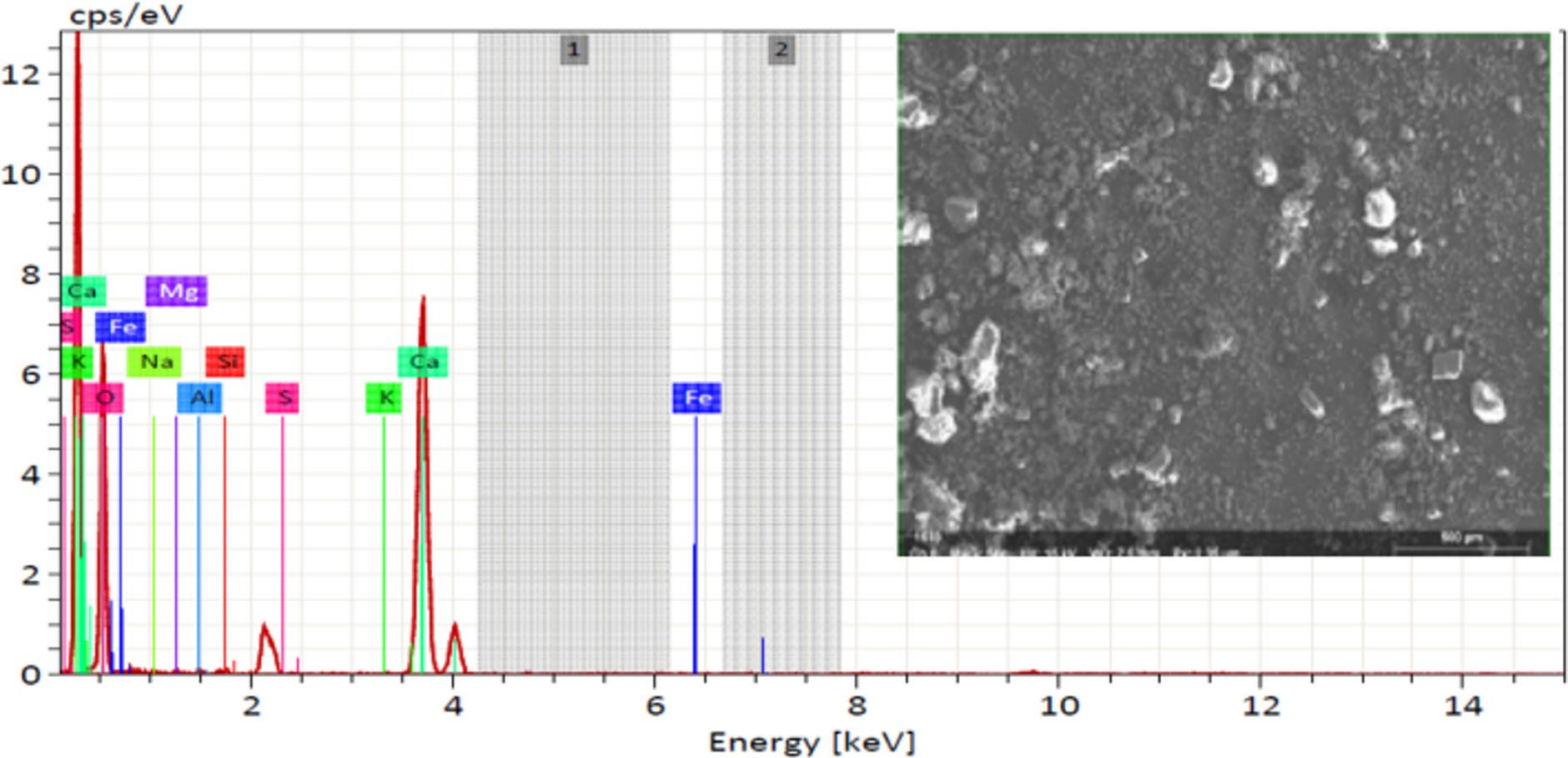
EDX analysis for MD.

**Fig. 5:**
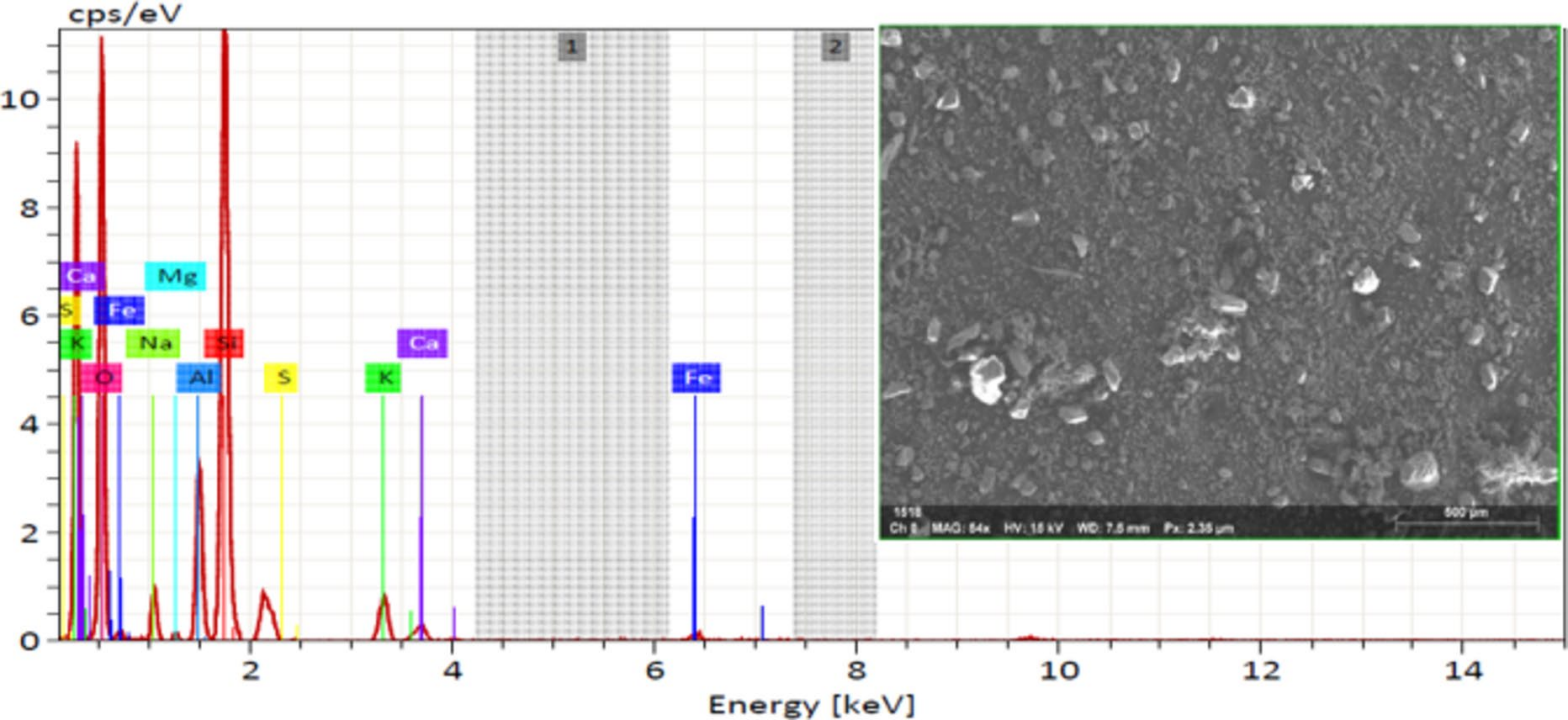
EDX analysis for GD.

**Fig. 6:**
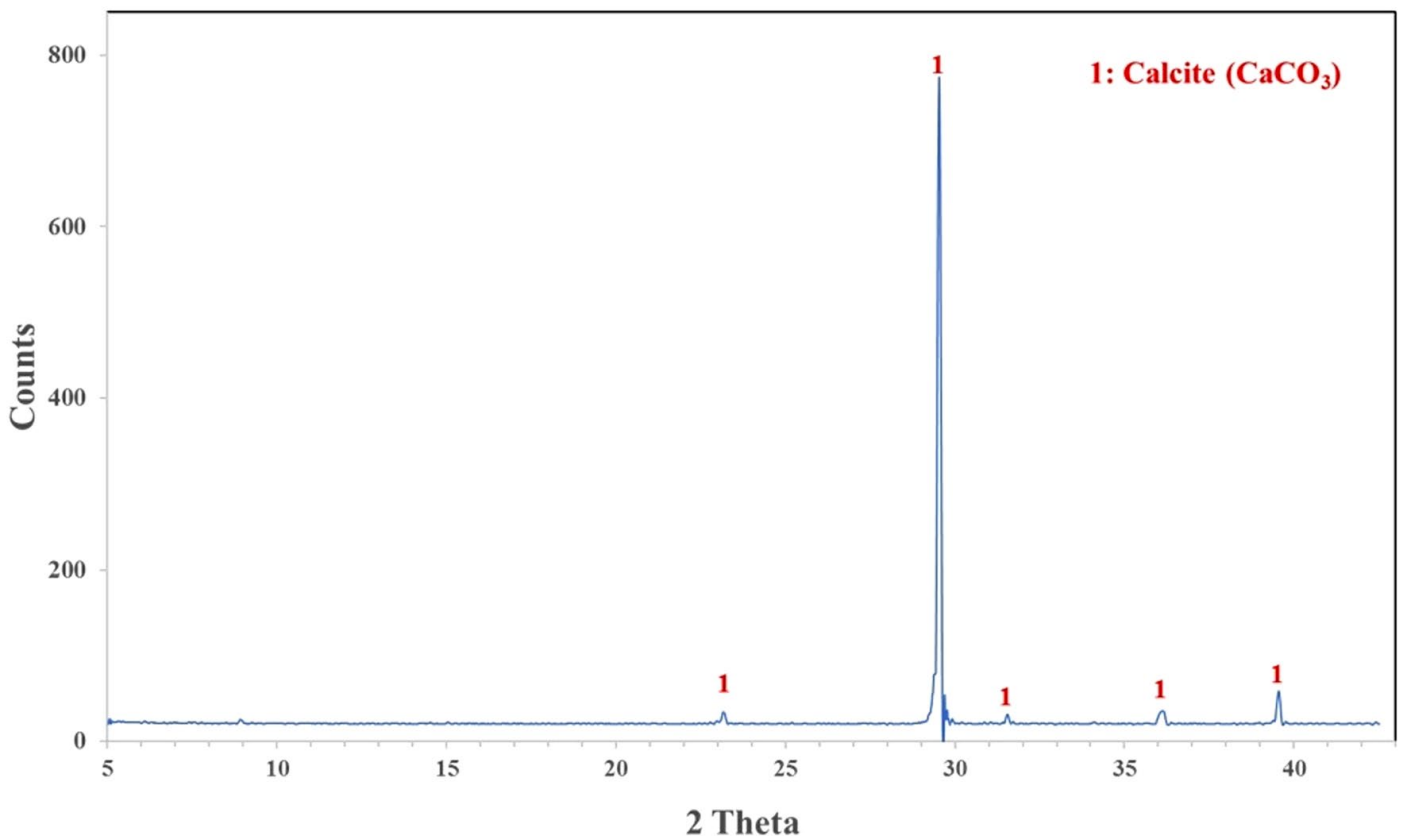
X-ray diffraction pattern for MD.

**Fig. 7:**
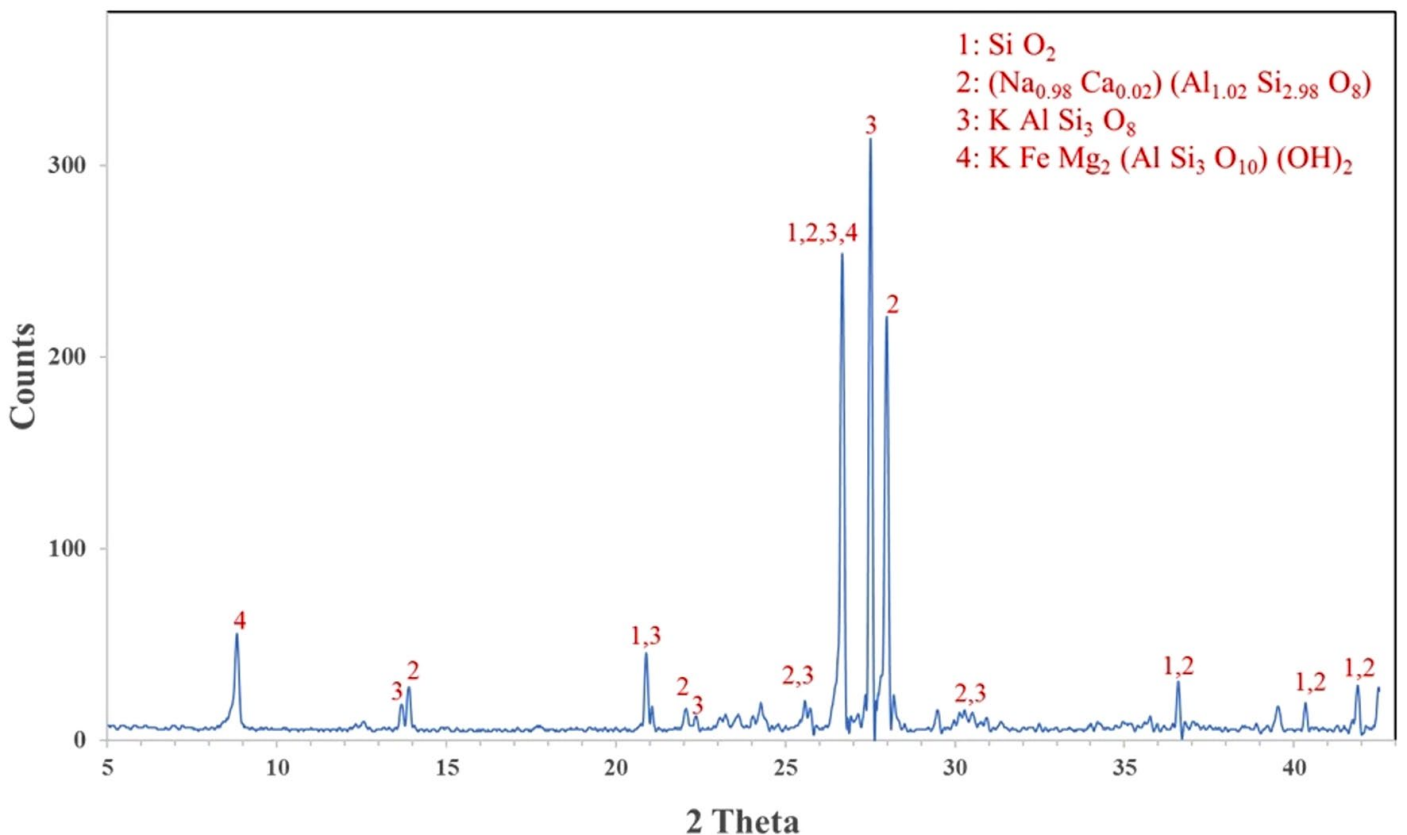
X-ray diffraction pattern for GD.

**Fig. 8:**
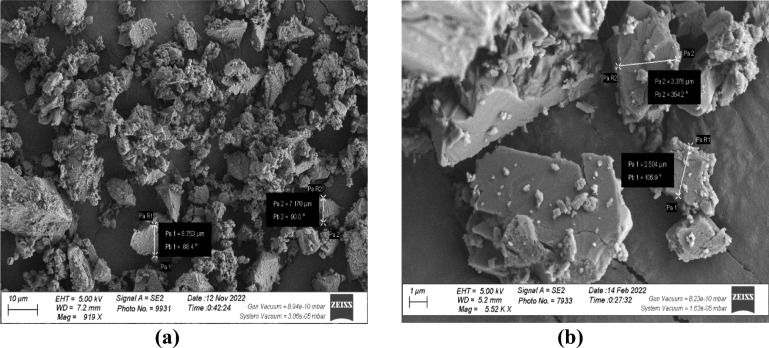
SEM images for (**a**) MD and (**b**) GD.

**Fig. 9:**
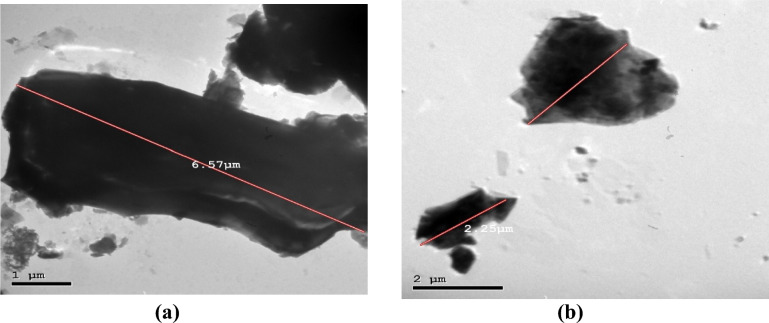
TEM images for (**a**) MD and (**b**) GD.

**Fig. 10:**
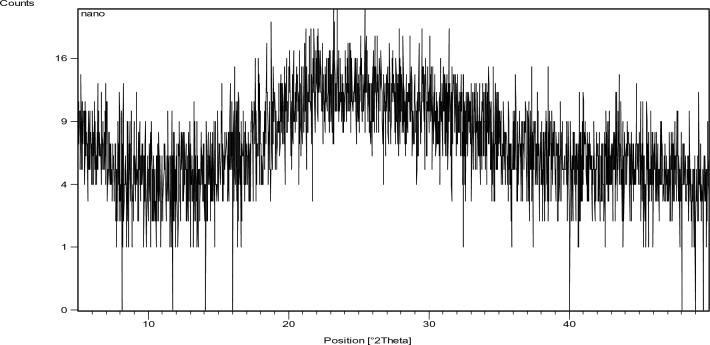
X-ray diffraction pattern of the used Nano alumina.

**Fig. 11:**
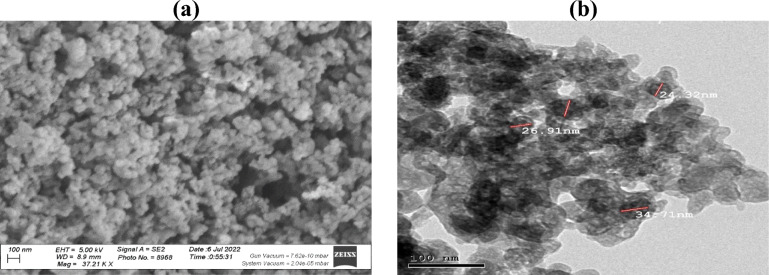
SEM and TEM of the used Nano alumina.

**Fig. 12:**
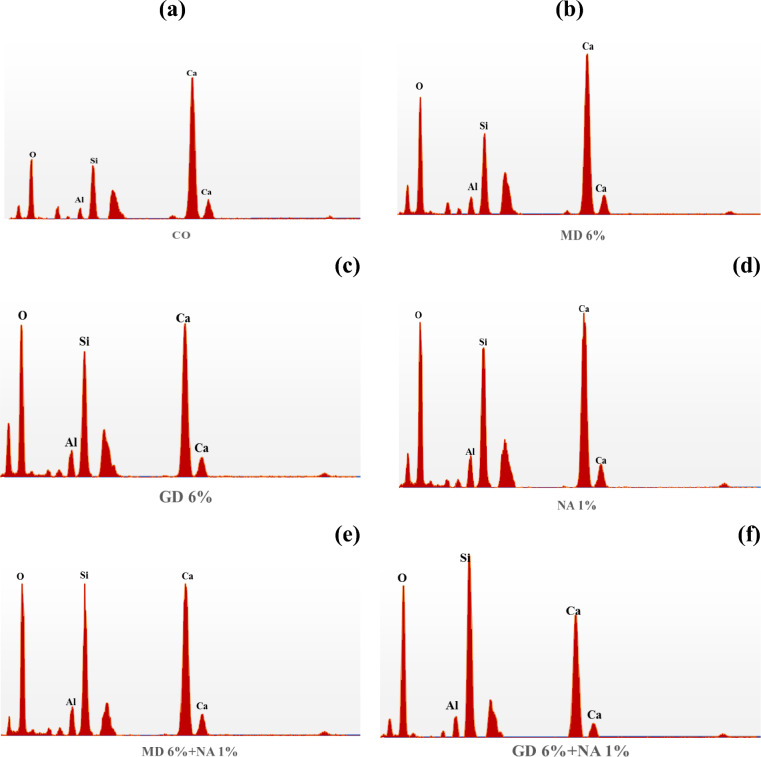
EDX patterns for (**a**) CO, (**b**) MD 6%, (**c**) GD 6%, (**d**) NA 1%, (**e**) MD6% + NA1% and (**f**) GD6% + NA1%.

XRF analysis was used to determine the chemical composition of MD and GD, and the results are presented in Table 1. On the other hand, the elemental composition that was determined through EDX analysis is shown in Table 2. The EDX technique is a method of analysis that allows for the chemical characterization and elemental analysis of various materials. EDX mappings were performed, and the results showed that the elemental distribution on the surface of materials was revealed. An EDX analysis with mapping was performed on the samples that were chosen, and the EDX patterns that were obtained are depicted in Figs. 4 and 5 respectively. GD is primarily composed of silicon dioxide SiO_2_ and aluminum oxide (Al_2_O_3_), whereas MD is primarily composed of calcium carbonates (CaCO_3_), as demonstrated by the results of the two techniques. In order to further characterize the waste materials that were utilized, techniques such as XRD, TEM, and SEM were utilized. These techniques were utilized to investigate the mineralogical composition, crystal structure, particle size, and microstructure of the dust. A comprehensive description of the marble and granite dust can be found in Figs. 6, 7, 8 and 9, which contains all of the relevant information. The XRF, XRD, SEM, and TEM techniques were utilized in order to investigate the chemical composition, mineralogical composition, crystalline structure, and microstructure of NA. The results of these analyses are presented in Table 1 and Figs. 10 and 11. Both the mix design of the investigated mixes and the elemental composition that was obtained through the EDX analysis of each mix are displayed in Tables 3, 4, and Fig. 12, respectively.

**Table 2:** EDX results of element compositions for marble and granite dust (% weight).

Element	O K	Ca K	Si K	Al K	Fe K	K K	Mg K	S K	Na K
Granite	29	69.590	0.300	0.260	–	0.110	0.370	0.210	0.150
Marble	47.470	2.230	31.690	7.780	2.650	4.660	0.340	0.120	3.050

**Table 3:** Concrete mix design (kg/m^3^).

Mix	Cement (kg)	Aggregate (kg)		Water (lit)	S.P (kg)	WMP (kg)	WGP (kg)	NAl (kg)
		Coarse	Fine					
Control mix	400	1080	635	160	6	–	–	–
MD 6%	376	1080	635	160	6	24	–	–
GD 6%	376	1080	635	160	6	–	24	–
NAl 1%	400	1080	635	160	6	–	–	4
MD6% + NA1%	376	1080	635	160	6	24	–	4
GD6% + NA1%	376	1080	635	160	6	–	24	4

**Table 4:** EDX results for different concrete mixes.

Element	Composition by (wt%)					
	CO	MD 6%	GD 6%	NA 1%	MD6% + NA1%	GD6% + NA1%
	Control mix	6% marble powder	6% granite powder	1% Nano alumina	6% marble powder + 1% Nano alumina	6% granite powder + 1% Nano alumina
O	38.490	44.190	44.960	42.620	41.160	47.220
Ca	44.040	35.600	28.610	30.800	37.600	26.640
Si	6.820	6.790	8.430	8.950	13.290	15.160
Al	1.300	1.460	1.740	1.890	2.380	1.820
Fe	1.580	1.800	1.790	1.820	1.800	2.370
Mg	0.220	0.680	0.500	0.580	0.160	0.370
Na	1.920	1.35	0.670	0.720	0.490	-
C	5.640	8.110	13.300	12.620	3.100	6.430
Density ρ(kg/m^3^)	2.420	2.620	2.650	2.510	2.690	2.720

### Radiation shielding performance

This study's concrete samples serve as the attenuator, and the attenuation factor measures the fraction of γ-ray photons passing through them. Table 5 displays the experimental and theoretical µ values of the prepared Concrete samples, as determined by using the MCS and PhyX software. The values computed by Phy and the simulated values of µ are very similar, with a maximum relative discrepancy of 4.168% ^65,66^. This study's concrete samples serve as the attenuator, and the attenuation factor measures the fraction of γ-ray photons passing through them. Table 5 displays the experimental and theoretical µ values of the produced Concrete samples, as determined by the MCS and Phy software. The decrease in the µ value occurs when the γ-energy levels increase, which is a trend that is consistently observed in all materials. This trend is particularly evident for the data where *µ* drops from 37.527 to 0.057 cm^−1^ for CO, from 34.796 to 0.060 cm^−1^ for MD, from 30.247 to 0.058 cm^−1^ for GD, and from 30.397 to 0.056 cm^−1^ for NA, from 38.866 to 0.063 cm^−1^ for MD + NA, and from 32.149 to 0.061 cm^−1^ for GD + NA at 0.015 ≤ Pγ ≤ 15 in MeVs.

**Table 5:** The prepared GMN-concrete samples' linear attenuation (µ) obtained using MCS and PhyX.

Energy, (MeV)	The linear attenuation (µ, cm^−1^)								
	CO			MD 6%			GD 6%		
	PhyX	MCS	Ώ%	PhyX	MCS	Ώ%	PhyX	MCS	Ώ%
0.015	37.942	37.527	1.105	35.200	34.796	1.160	30.611	30.247	1.204
0.030	5.354	5.335	0.348	5.023	5.005	0.366	4.419	4.402	0.380
0.050	1.482	1.446	2.490	1.438	1.401	2.614	1.310	1.275	2.714
0.080	0.641	0.618	3.824	0.655	0.629	4.015	0.627	0.602	4.168
0.100	0.495	0.480	3.305	0.517	0.500	3.470	0.505	0.487	3.602
0.200	0.314	0.307	2.150	0.338	0.331	2.258	0.340	0.332	2.344
0.300	0.263	0.259	1.281	0.285	0.281	1.345	0.287	0.283	1.397
0.400	0.232	0.231	0.574	0.252	0.251	0.603	0.255	0.253	0.626
0.500	0.211	0.210	0.333	0.229	0.229	0.349	0.232	0.231	0.363
0.600	0.195	0.194	0.587	0.212	0.210	0.616	0.214	0.213	0.640
0.800	0.171	0.170	0.168	0.186	0.185	0.176	0.188	0.187	0.183
1	0.153	0.153	0.101	0.167	0.166	0.106	0.169	0.168	0.110
2	0.108	0.108	0.209	0.117	0.117	0.219	0.118	0.119	0.228
3	0.089	0.089	0.519	0.096	0.096	0.545	0.097	0.097	0.566
4	0.078	0.079	0.749	0.084	0.085	0.786	0.085	0.085	0.816
5	0.071	0.072	0.702	0.077	0.077	0.737	0.077	0.078	0.765
6	0.067	0.068	0.593	0.072	0.072	0.622	0.072	0.072	0.646
8	0.062	0.062	0.534	0.066	0.066	0.560	0.066	0.066	0.582
10	0.059	0.060	0.510	0.063	0.063	0.536	0.062	0.062	0.556
15	0.057	0.057	0.494	0.059	0.060	0.519	0.058	0.058	0.539

Energy, (MeV)	The linear attenuation (µ, cm^−1^)								
	NA 1%			MD6% + NA1%			GD6% + NA1%		
	PhyX	MCS	Ώ%	PhyX	MCS	Ώ%	PhyX	MCS	Ώ%
0.015	30.736	30.397	1.116	39.292	38.866	1.094	32.487	32.149	1.049
0.030	4.415	4.400	0.352	5.561	5.542	0.345	4.670	4.655	0.331
0.050	1.291	1.259	2.515	1.563	1.525	2.465	1.374	1.342	2.365
0.080	0.606	0.584	3.862	0.694	0.668	3.786	0.651	0.628	3.633
0.100	0.484	0.469	3.338	0.542	0.525	3.272	0.522	0.506	3.140
0.200	0.323	0.316	2.172	0.349	0.342	2.129	0.349	0.342	2.043
0.300	0.272	0.269	1.294	0.293	0.289	1.269	0.295	0.291	1.217
0.400	0.242	0.240	0.580	0.259	0.258	0.569	0.262	0.260	0.546
0.500	0.220	0.219	0.336	0.236	0.235	0.329	0.238	0.237	0.316
0.600	0.203	0.202	0.593	0.217	0.216	0.581	0.220	0.218	0.558
0.800	0.178	0.177	0.169	0.191	0.190	0.166	0.193	0.192	0.159
1	0.160	0.159	0.102	0.171	0.171	0.100	0.173	0.173	0.096
2	0.112	0.112	0.211	0.120	0.121	0.207	0.122	0.122	0.198
3	0.092	0.092	0.525	0.099	0.099	0.514	0.099	0.100	0.493
4	0.080	0.081	0.756	0.087	0.088	0.741	0.087	0.088	0.711
5	0.073	0.074	0.709	0.080	0.080	0.695	0.079	0.080	0.667
6	0.068	0.069	0.599	0.075	0.075	0.587	0.074	0.075	0.563
8	0.062	0.063	0.539	0.069	0.069	0.528	0.068	0.068	0.507
10	0.059	0.060	0.515	0.066	0.066	0.505	0.064	0.065	0.485
15	0.056	0.056	0.499	0.062	0.063	0.489	0.060	0.061	0.470

**Fig. 13:**
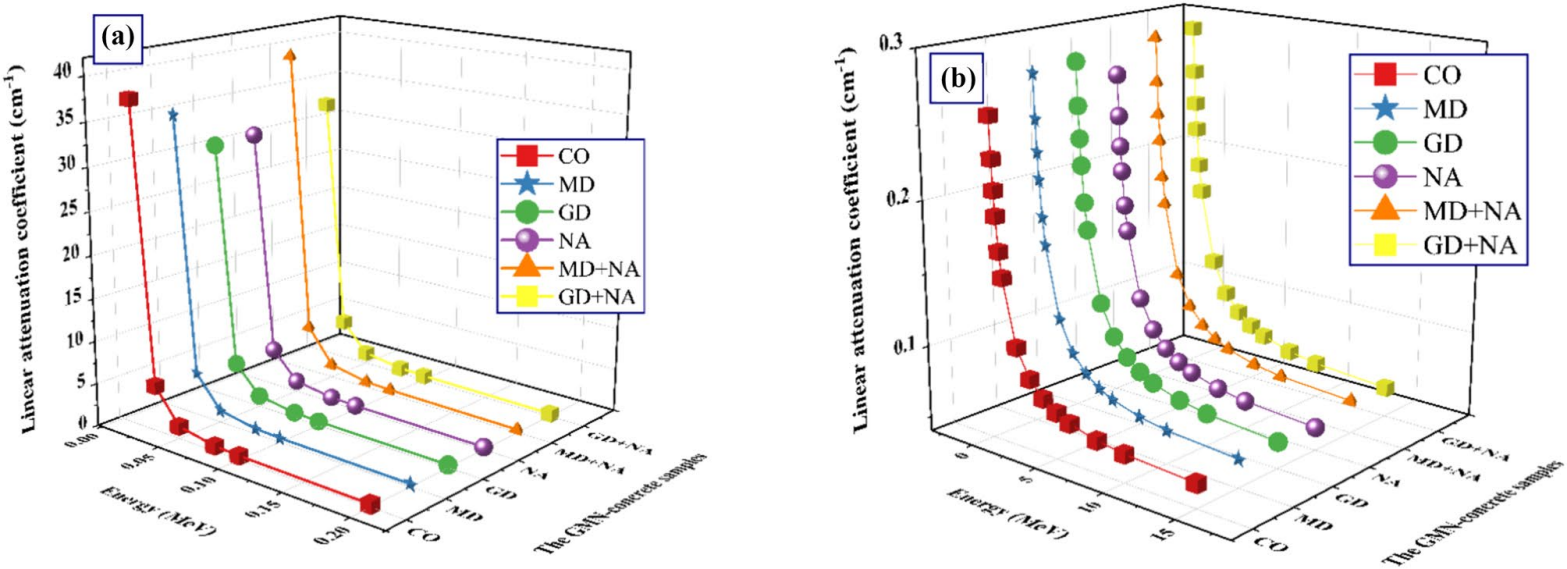
Influence of γ-ray energy on linear attenuation of (**a**) photoelectric, (**b**) Compton scattering processes of the prepared MGN-concretes.

As realized in Fig. 13a, there is a substantial decrease in the *µ* for the synthetic GMN-concrete samples due to the PEE, which σ α ${1/P}_{\gamma }^{4:5}$. The enrichment of Pγ led to a decrease in the σ of interactions, which in turn led to a decrease in the γ-electron interactions and values. The enhancement of the Pγ at in MeVs; 0.015 ≤ Pγ ≤ 0.200 causes a solid exponential decreasing trend from 37.527 to 0.307 cm^−1^ for CO, from 34.796 to 0.331 cm^−1^ for MD, from 30.247 to 0.332 cm^−1^ for GD, and from 30.397 to 0.316 cm^−1^ for NA, from 38.866 to 0.342 cm^−1^ for MD + NA, and from 32.149 to 0.342 cm^−1^ for GD + NA concrete sample.

Moreover, as shown in Fig. 13b, the values of Pγ at energies ranging from 0.300 to 15 MeV show an exponential decline as the energy exceeds 0.200 MeV. The interaction between COMSE and variations in σ α 1/Pγ ^67–69^. Is responsible for the exponential drop. Higher Pγ interacting with the atoms of the material is less likely due to their incredibly high velocity, which explains this phenomenon. Therefore, the likelihood of γ-absorption diminishes and the likelihood of γ-scattering increases as Pγ increases. As the number of γ-electron interactions decreased, the σ gradually decreased as well, and the µ values gradually fell after the Pγ values increased. The reduction in the µ values was from 0.259 to 0.057 cm^−1^ for CO, from 0.281 to 0.060 cm^−1^ for MD, from 0.283 to 0.058 cm^−1^ for GD, and from 0.269 to 0.056 cm^−1^ for NA, from 0.289 to 0.063 cm^−1^ for MD + NA, and from 0.291 to 0.061 cm^−1^ for GD + NA at in MeVs 0.300 ≤ Pγ ≤ 15.

Due to the high densities of the GD + NA and MD + NA (2.720 and 2.690 kg m^−3^), the high sufficient atomic number of the elements of Si, Al, Fe, etc., and the nano-alumina doping (1% wt.), GD + NA and MD + NA often exhibit the most excellent µ values compared to the other concrete samples.

**Fig. 14:**
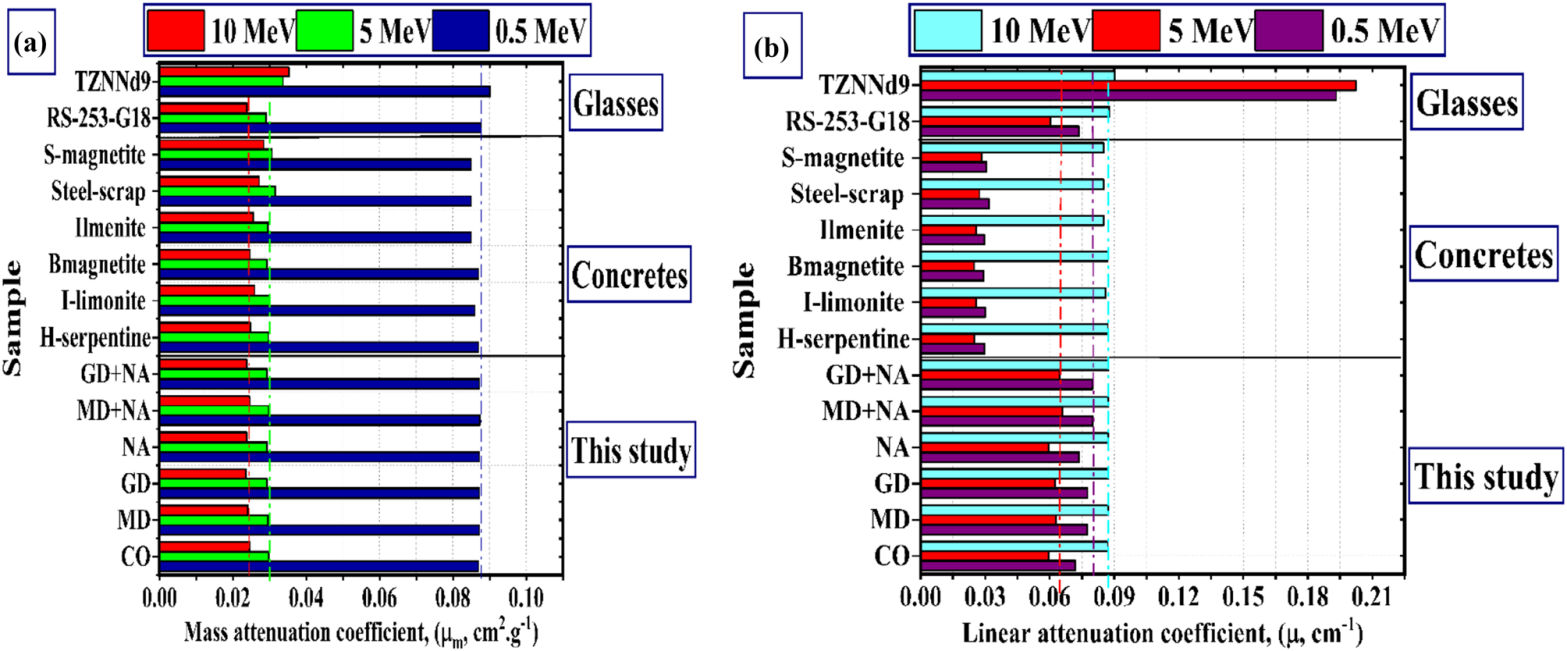
(**a**) The mass attenuation (µm, cm^2^ g^−1^), and (**b**) The linear attenuation (µ, cm^−1^) for the MGN-concrete samples with reference concert and glass samples.

Figure 14a displays a comparison of the µ_m_ between samples of GMN-concrete, commercial concrete, and a few glasses. We will be comparing energies of 0.5, 5, and 10 MeV ^70^. In comparison to the concrete and glass samples, as well as the TZNNd9 glass sample, the µ values of the GMN-concrete samples are higher. As can be observed in Fig. 14b, the comparison with respect to the µ yields identical results.

**Fig. 15:**
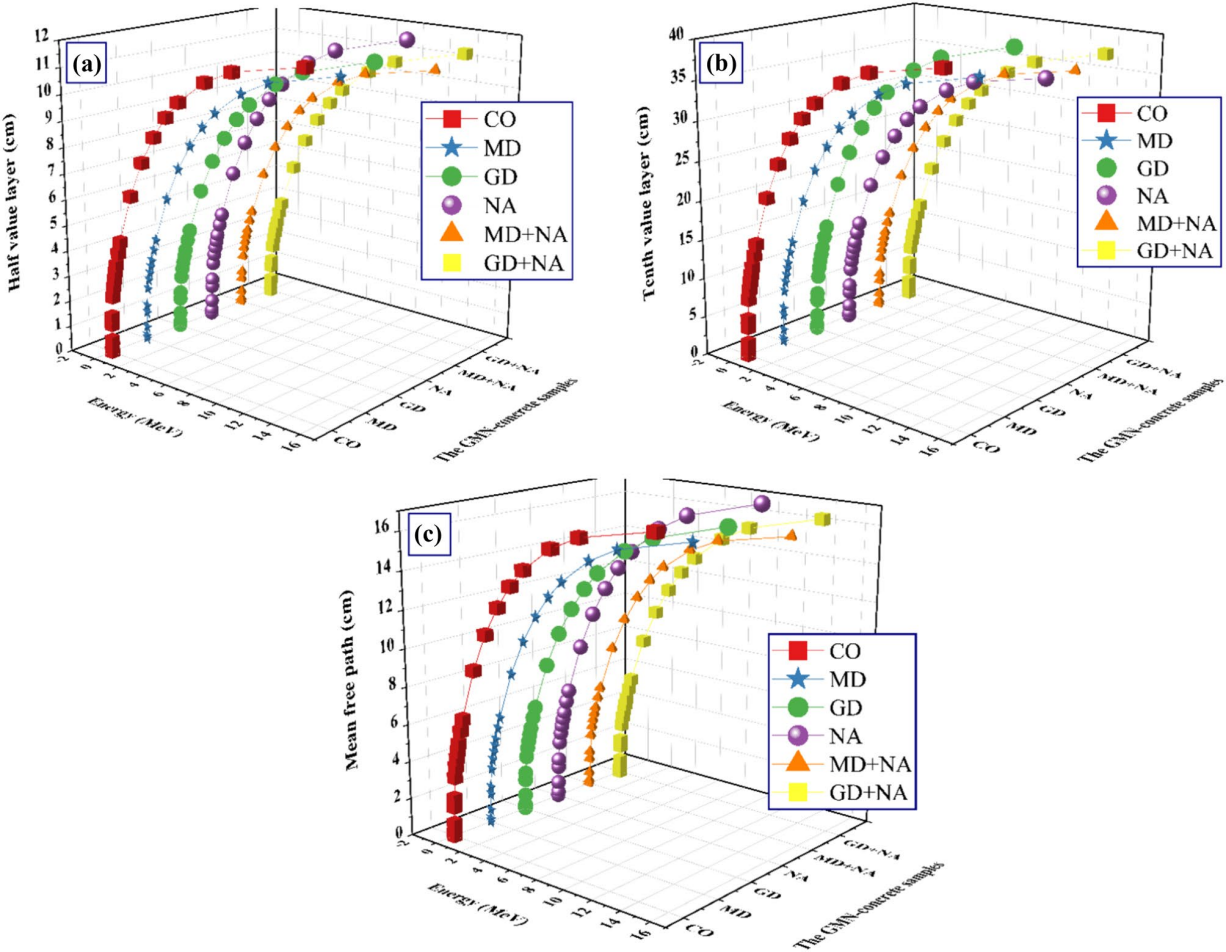
(**a**) The half value layer (H_1/2_), (**b**) The Tenth value layer (T_1/10_), and the mean free path (MFp) for the prepared GMN-concretes versus the γ-energy.

The half-value layer thickness (H_1/2_), the tenth-value layer thickness (T_1/10_), and the mean free path (MFp) are three common methods that are utilized to quantify the effectiveness of radiation shielding ^71–75^. This demonstrates the thickness of the shielding material as well as whether or not it is able to block radiation. By decreasing either value, the radiation shielding performance is improved for a given photon energy. This is because radiation is attenuated through a narrower zone when the value is decreased. The H_1/2_ of the synthesized Concrete samples increased as the µ decreased_._ The H_1/2_ values grew from 0.018 to 12.199 cm for CO, from 0.020 to 11.634 cm for MD, from 0.023 to 11.863 cm for GD, and from 0.023 to 12.382 cm for NA, from 0.018 to 11.044 cm for MD + NA, and from 0.022 to 11.404 cm for GD + NA sample with raising at 0.015 ≥ Pγ ≥ 15 MeV (Fig. 15a**)**. The values of the T_1/10_ follow the same pattern as the H_1/2_ as demonstrated in Fig. 15b. Figure 15c represents the *MFp* of the examined Concrete samples as it varies with energy. The *MFp* values were found from 0.027 to 17.600 cm for CO, from 0.029 to 16.785 cm for MD, from 0.033 to 17.114 cm for GD, and from 0.033 to 17.863 cm for NA, from 0.026 to 15.933 cm for MD + NA, and from 0.031 to 16.453 cm for GD + NA sample.

**Fig. 16:**
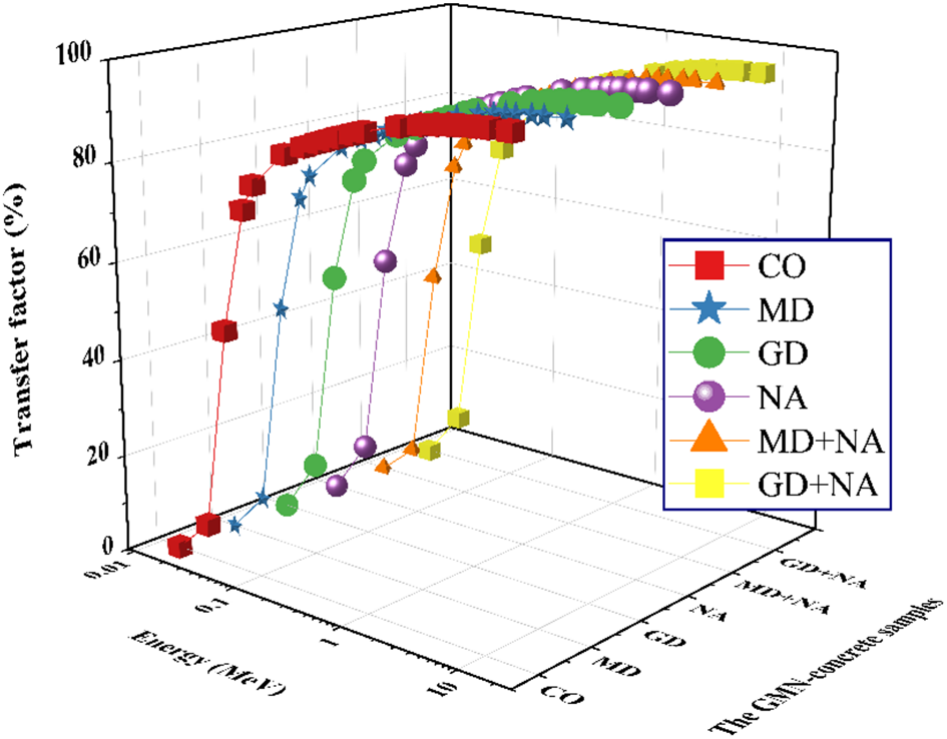
The transfer factor (GMN_TF_) for the prepared GMN concrete samples against photon energy.

**Fig. 17:**
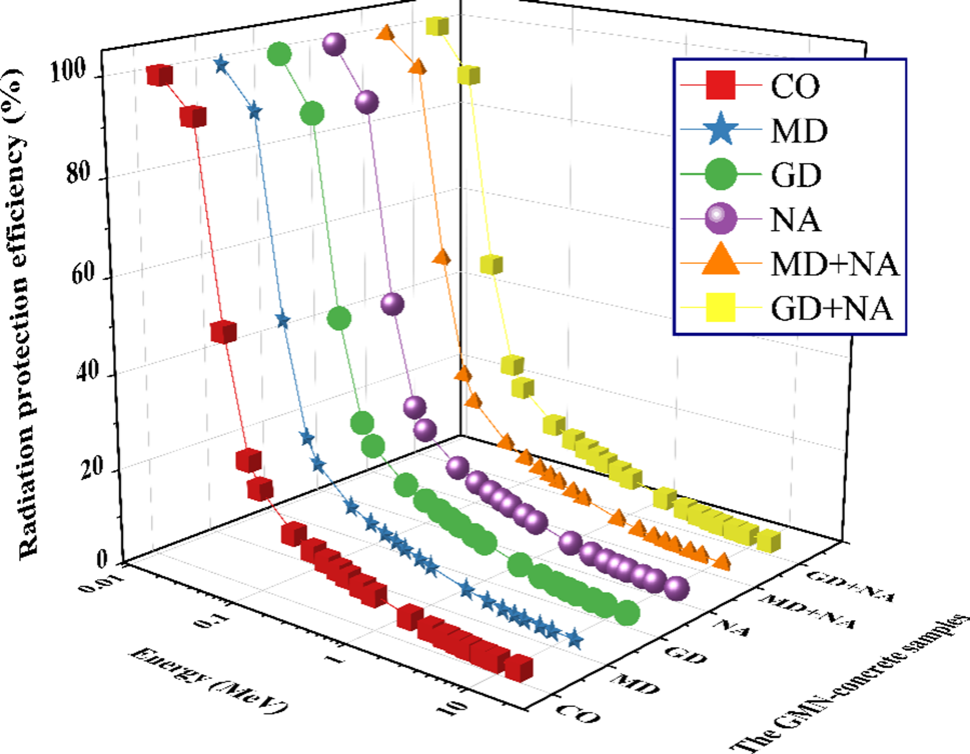
The radiation protection efficiency (GMN_RPE_) for the prepared GMN concrete samples versus photon energy.

The effects of γ-ray energy and the concentration doping of Waste Granite, Marble, and Nano-Alumina on the GMN_TF_ and GMN_RPE_ for the tested concrete are shown in Figs. 16 and 17, respectively. These figures clearly show it. At 0.015 MeV, which is the shallow γ-ray energy, the RPE values are almost 100%. A considerable drop in GMN_RPE_ levels was seen as a result of an increase in both the γ-energy and the penetrating strength of the supplied γs. Hence, γ-electron interactions inside the produced concretes are reduced as the γ-energy is increased. Reducing the γ-electron interaction leads to an increase in the quantity of scattered γs, which negatively impacts the GMN_RPE_ for the GMN concrete samples.

For, the GMN_RPE_ values dropped from ≈ 100 at 0.015 MeV for all GMN concrete samples to 14.240%, 15.248%, 15.297%, 14.609%, 15.704%, and 15.732%, respectively, for CO, MD, GD, NA, MD+NA, and GD+NA samples at γ-ray energy of 0.200 MeV. The results confirm a great shielding capacity for the GMN concrete samples from 0.015 ≤ γ-energy ≤ 0.200 MeV.

**Fig. 18:**
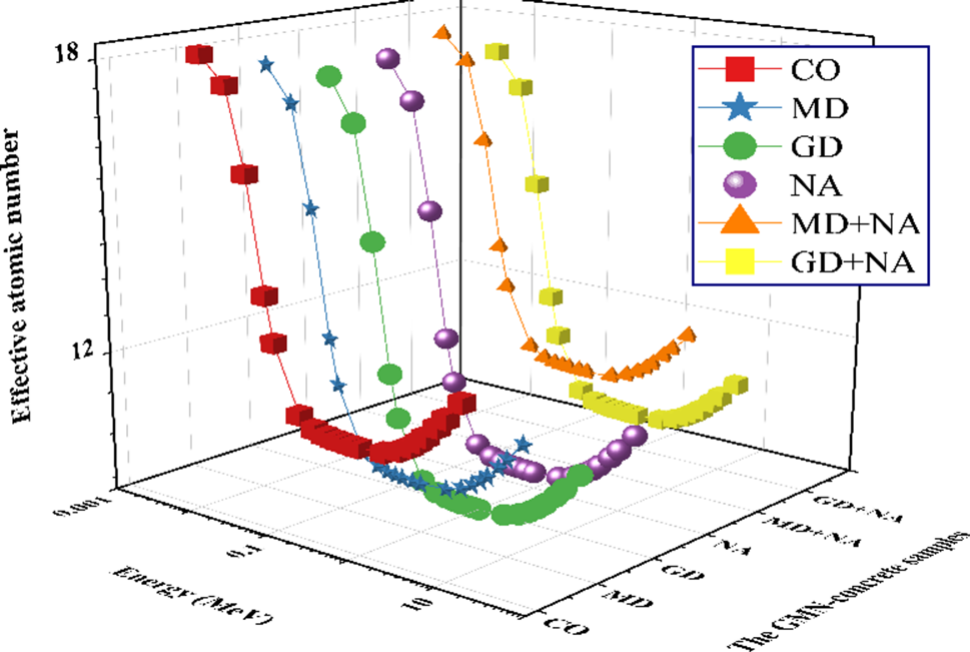
The effective atomic number (GMN_Zeff_) for the prepared GMN-concrete samples vs. photon energy.

Figure 18 shows graphs of the adequate atomic number for the GMN-concrete samples (GMN_zeff_) vs γ-energy ranging from 0.015 ≥ Pγ ≥ 15 MeV for the examined glasses. Higher MGN_zeff_ values imply a more favorable radiation interaction, particularly in the COMSE zone. To shield from high- Pγ energy, materials with higher GMN_zeff_ value might be better ^76^. For the studied materials, the GMN_zeff_ values decrease as the MeVs increase. The range of GMN_zeff_ for the glasses varied for the energy spectra of interest, having a range of from 18.376 to 12.523 cm for CO, from 17.900 to 11.526 cm for MD, from 17.372 to 10.725 cm for GD, and from 17.560 to 11.008 cm for NA, from 17.947 to 12.362 cm for MD+NA, and from 17.287 to 11.224 cm for GD+NA concrete sample. In light of this, it is possible to draw the conclusion that the radiation shielding efficiency of materials shifts in accordance with the energy of the radiation; it is possible that one substance functions more effectively at higher or lower MeVs than another.

**Fig. 19:**
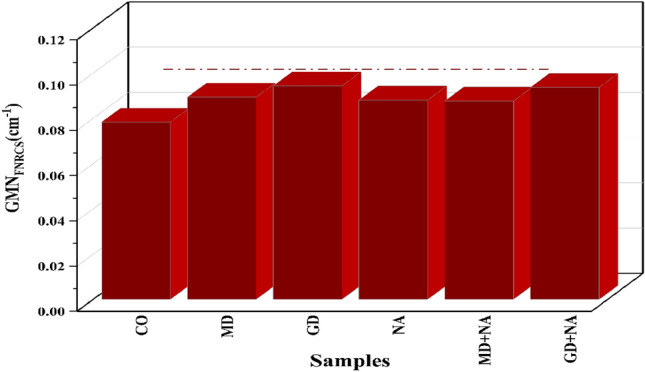
The fast neutron removal cross-section (GMN_FNRCS_) for the prepared GMN-concretes.

**Fig. 20:**
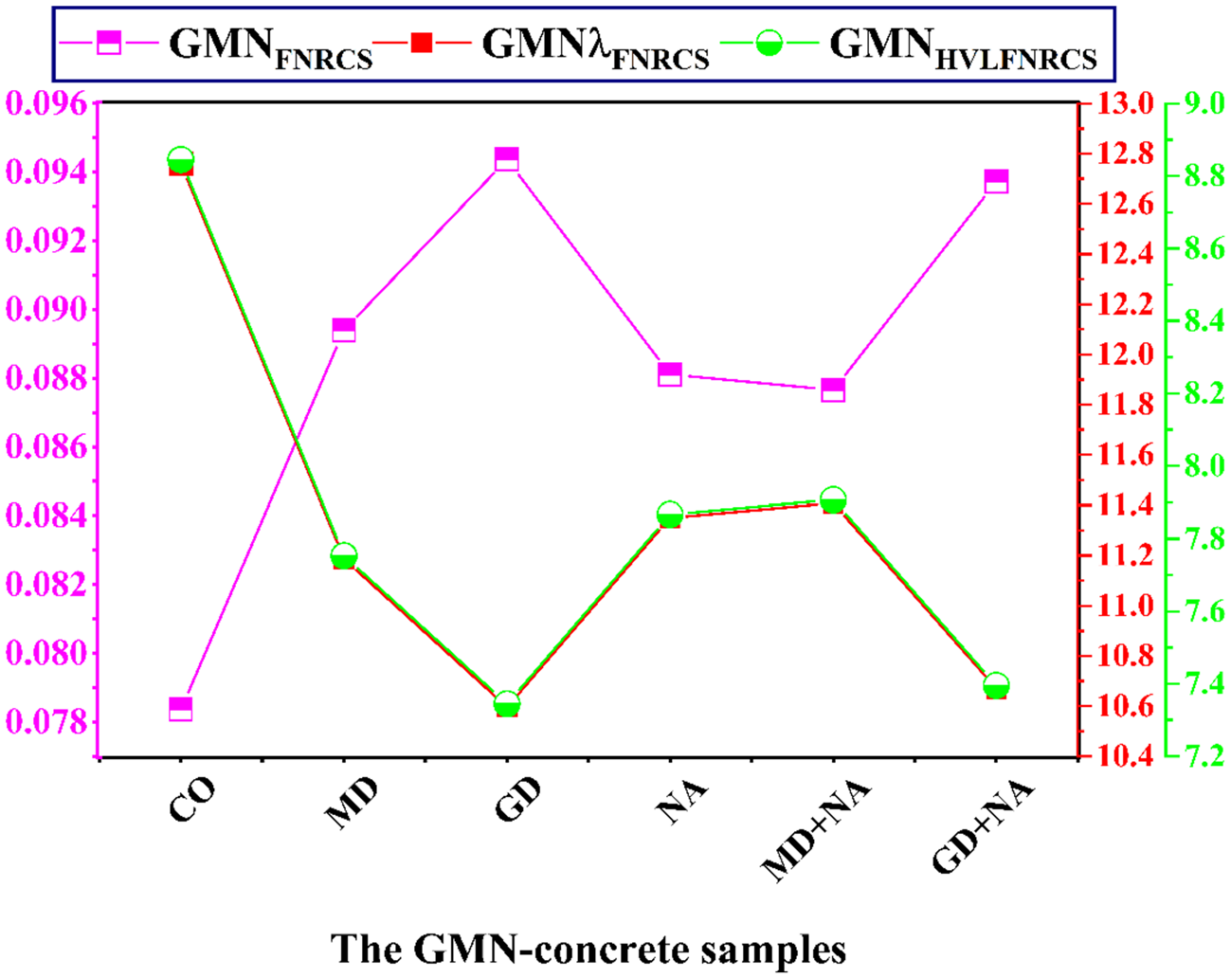
The prepared GMN-concrete samples' fast neutron removal cross-section (GMN_FNRCS_), half-value layer (GMN_HVLFNRCS_), and relaxation length (GMN_λFNRCS_).

The results show that the fast neutron removal cross-section (GMN_FNRCS_) for the GMN-concrete samples were 0.078 cm^−1^, 0.089 cm^−1^, 0.094 cm^−1^, 0.088 cm^−1^, 0.088 cm^−1^, and 0.094 cm^−1^ for CO, MD, GD, NA, MD+NA, and GD+NA correspondingly. The GD+NA had the most efficient GMN_FNRCS_, primarily because of its elevated concentration of light elements (oxygen) and its high density (2.720) (Fig. 19). The GMN_HVLFNRCS_ and GMNλ_FNRCS_ values for the synthetic concrete samples are likewise displayed (Fig. 20) ^70^. The HVL_FNRCS_ and λ_FNRCS_ values for the GD+NA sample were the lowest, according to the simulated GMN_FNRCS_ values. The GMN_FNRCS_ values of the synthesized GD+NA samples were higher than those prepared. Therefore, we can conclude that the six GMN-concrete systems under investigation have good neutron shielding properties.

## Conclusion

This work examines the γ-ray and neutron shielding properties of six mixes of waste Granite, Marble, and Nano-Alumina additives on ordinary concretes (GMN-concrete). The ${\text{GMN}}\mu$ order is CO < MD < GD < NA < MD + NA < GD + NA. The MD + NA and GD + NA samples have the lowest H_1/2,_ T_1_/_10_, and MFp. Within the investigated Pγ, GMN_zeff_ changes: from 18.376 to 12.523 for CO, from 17.900 to 11.526 for MD, from 17.372 to 10.725 for GD, and from 17.560 to 11.008 for NA, from 17.947 to 12.362 for MD + NA, and from 17.287 to 11.224 for GD + NA concrete sample. The GMN_FNRCS_ of the GMN-concrete samples have values ranging from 0.076 to 0.094 cm^−1^ for the CO, MD, GD, NA, MD + NA, and GD + NA concretes. In light of this, it can be concluded that the GMN-concrete samples that were studied offer the maximum level of protection against γ-rays and fast neutrons. The exceptional performance of combinations of waste marble, granite, and nano-Alumina on conventional concretes has made it possible for radiation shielding in nuclear and medical facilities to become a reality. This is the last but not the least of the potential achievements.

## Ethical approval

This article doesn't contain any studies involving animals performed by any authors. Also, this article does not contain any studies involving human participants performed by authors.

## Consent to participate

All Authors agree to participate in the published version of the manuscript.

## Data Availability

All data generated or analyzed during this study are included in this published article.
